# Association between problematic smartphone use and mental health among high school students in southern Brazil

**DOI:** 10.1590/1984-0462/2026/44/2026001

**Published:** 2026-11-09

**Authors:** Bruno Pedrini de Almeida, Rafael Clain Martins, Laís Werneck Azevedo, Eduardo Gauze Alexandrino, Pâmela Moraes Volz, Michael Pereira da Silva, Samuel Carvalho Dumith

**Affiliations:** a Universidade Federal do Rio Grande, Rio Grande, RS, Brazil.

**Keywords:** Smartphone addiction, Mental health, Depression, Anxiety, Psychological stress, Students, Smartphone, Saúde mental, Depressão, Ansiedade, Estresse psicológico, Estudantes

## Abstract

**Objective:**

To investigate the association between problematic smartphone use (PSU) and mental health symptoms among high school students in the south of Brazil.

**Methods:**

A cross-sectional study was conducted in 2022 to assess mental health symptoms using the Depression Anxiety Stress Scale. PSU was assessed using the Smartphone Addiction Scale – Short Version, with scores ≥33 indicating PSU. Dichotomous variables were created to assess the presence of symptoms of depression, anxiety, and stress. Associations were tested using Fisher’s exact test and Poisson regression with robust variance.

**Results:**

Most participants were boys (55.2%), and 52.5% aged ≥18 years. PSU prevalence was 35.5%, while depression, anxiety, and stress symptoms had prevalence values of 37.1, 44.7, and 45.7%, respectively, and 62.7% of the students exhibited at least one symptom. Among students with PSU, the proportion of symptoms was significntly higher: 44.8% for anxiety (p=0.017), 57.1% for depression (p<0.001), and 58.2% for stress (p<0.001). As per the adjusted analysis, students with PSU had higher prevalence of depression (prevalence ratio [PR] 1.26; 95% confidence interval [CI] 1.02–1.56) and stress (PR 1.34; 95%CI 1.09–1.64) symptoms, and at least one of the three symptoms (PR 1.24; 95%CI 1.08–1.43), when compared to those without PSU.

**Conclusions:**

In this study, PSU was associated with higher prevalence of mental health symptoms among high school students in the south of Brazil. These results provide evidence that can guide clinical practice and public health policies aimed at promoting mental health.

## RESUMO

**Objetivo:**

Investigar a associação entre o uso problemático de *smartphone* (UPS) e sintomas de saúde mental em estudantes do ensino médio do sul do Brasil.

**Métodos:**

Foi realizado um estudo transversal em 2022, no qual avaliaram-se sintomas de saúde mental usando a *Depression Anxiety Stress Scale* e o UPS pela *Smartphone Addiction Scale – Short Version,* em que pontuações ≥33 determinam UPS. Variáveis dicotômicas foram criadas para verificar a presença de sintomas de depressão, ansiedade e estresse. Associações foram testadas pelo teste exato de Fisher e pela regressão de Poisson com variância robusta.

**Resultados:**

A maioria da amostra foi composta por meninos (55,2%), e 52,5% dela tinha idade ≥18 anos. O UPS apresentou prevalência de 35,5%, enquanto sintomas de depressão, ansiedade e estresse apresentaram 37,1, 44,7 e 45,7%, respectivamente, e 62,7% dos estudantes apresentaram pelo menos um sintoma. Entre estudantes com UPS, a proporção de sintomas foi significativamente superior: 44,8% para ansiedade (p=0,017), 57,1% para depressão (p<0,001) e 58,2% para estresse (p<0,001). De acordo com a análise ajustada, estudantes com UPS tiveram maior prevalência de depressão (razão de prevalências [RP] 1,26; intervalo de confiança [IC] 95% 1,02–1,56), estresse (RP 1,34; IC95% 1,09–1,64) e pelo menos um sintoma (RP 1,24; IC95% 1,08–1,43), quando comparados àqueles sem UPS.

**Conclusões:**

O UPS apresentou associação significativa com sintomas de saúde mental em estudantes no ensino médio do sul do Brasil. Esses resultados fornecem evidências que podem orientar práticas clínicas e políticas de saúde pública voltadas para a promoção da saúde mental.

## INTRODUCTION

The widespread use of smartphones has reshaped the technological landscape over the past decades.^1^ Due to its diverse range of applications, ranging from productivity tools to entertainment options, with increasingly easy portability, improved functionality, and a focus on ease of use,^2^ smartphone use has become widely integrated into everyday life.^3^

The popularity of this device is evidenced according to data from 2024, indicating that around 60% of the world’s population (4.8 billion people) owned a smartphone, a figure likely to increase in thecoming years.^4^ In a nationwide survey, it was found that the Brazilian scenario substantially contributes to this new reality observed around the world, with 91.9% of households having access to a smartphone.^5^

Although many benefits of smartphone use and its versatility in everyday life are highlighted, there are concerns about its maladaptive use. In this regard, the literature has highlighted that problematic smartphone use (PSU), characterized as a behavioral disorder,^6^ appears to result in adverse health effects, especially among adolescents and young people,^7,8^ with robust evidence pointing to an increased likelihood of depression, anxiety, and perceived stress for those with PSU.^9^

Given that mental health disorders, such as depression, anxiety, and stress, are the leading cause of disability worldwide, the World Health Organization has issued warnings regarding the deleterious impacts of mobile device use on the psychological well-being of young people.^10,11^ Nevertheless, scientific evidence within the Brazilian context is scarce despite the high prevalence and projected growth of smartphone usage in the country. Except for the study conducted by Nunes et al.,^12^ which investigated common mental disorders without specifying the mental health symptoms, empirical data remains limited.

Thus, in the present study, we aimed to investigate the association between PSU and mental health symptoms among students in the south of Brazil.

## METHOD

A cross-sectional study was conducted with high school students from a federal educational institute in the municipality of Rio Grande, in the state of Rio Grande do Sul, in the extreme south of Brazil. This study is derived from the School Health Program, which aims to map the health profile of high school students, and was approved by the Research Ethics Committee of Universidade Federal do Rio Grande (No. 26359019.00000.5324). The STROBE statement was used to guide the manuscript.

The institute had 480 students enrolled in the 1st to 3rd years of high school, and all were invited to participate in the study, drawing a convenience sample. Students were considered eligible if they regularly attended Physical Education classes (at least 75% regular attendance), and ineligible if they had physical and/or cognitive impairments that prevented them from participating in the study. Attendance at Physical Education classes was included as an eligibility criterion, as the designated data collection area is adjacent to the gymnasium where those classes are held. Students who chose not to participate were considered refusals, and those who could not be found after four attempts to collect data were considered losses. All participants signed an Informed Assent Form agreeing to participate in the study, and their parents or guardians signed an Informed Consent Form. This process is summarized in the flowchart of participants’ selection (Figure 1).

**Figure 1:**
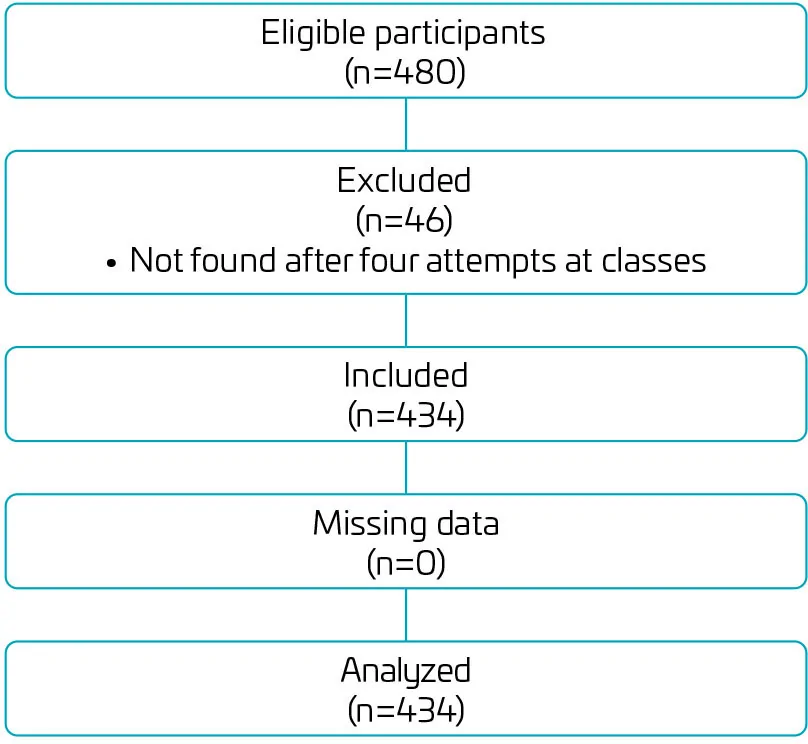
STROBE flowchart of participants’ selection.

Source: Created by the authors.

Data were collected between October and November 2022 during Physical Education classes, for logistic reasons. The time required to complete the questionnaires ranged from 20 to 30 minutes. Participants used self-administered questionnaires developed and stored on the Research Electronic Data Capture (REDCap) platform and answered them individually during interviews on tablets.

The study outcomes were assessed using the short version of the Depression Anxiety Stress Scale (DASS-21), which is validated for use in Brazil and demonstrated adequate internal consistency (Depression=0.90; Anxiety=0.83; Stress=0.86).^13^ The DASS-21 contains 21 items for assessing depression, anxiety, and stress (seven items for each domain). Each item has a score ranging from 0 (no symptom) to 3 (worst symptom), according to the degree of agreement with the statement (0=did not happen to me this week; 3=happened to me most of the time). Based on these scores, cutoff points were adopted considering the average score to classify the severity of each mental health outcome as follows: Normal≤2.34; Mild=2.34 to 2.61; Moderate=2.61 to 2.85; Severe=2.85 to 2.94; and Extremely severe≥2.94.^14^ For analytical purposes, dichotomous variables were created to indicate the presence of symptoms for each outcome, grouped as follows: No symptoms (normal) vs. Symptoms present (mild, moderate, severe, or extremely severe).

PSU was assessed using the Smartphone Addiction Scale – Short Version (SAS-SV), which is validated for use in Brazil and has adequate reliability (α=0.81).^15^ On this scale, each of the ten questions investigating PSU presents response options ranging from 1 (strongly disagree) to 6 points (strongly agree), such as: “I use my mobile phone more than I intended” or “I have difficulty concentrating during classes, while performing tasks or working due to mobile phone use.” Thus, the total score ranges from 10 to 60 points, and participants with a score ≥33 are classified as having PSU regardless of sex.^15^

Information was collected on sex (boys/girls), age (in complete years), skin color (white, Black, Asian, and mixed-race — grouped as white/other for further analysis), and socioeconomic status (SES). SES was assessed using the number of consumer goods reported in the participants’ households. A “household asset index” was created based on items that had a coefficient greater than 0.20 in the covariance matrix, whose highest covariance coefficients were: number of air conditioners (0.52), number of bathrooms in the home (0.47), and number of cars (0.44). This variable was split into quartiles.

The physical activity (PA) indicator was calculated as the total time spent engaging in physical activity during the week prior to the survey, accounting only for extracurricular activities and excluding travel between home and school and Physical Education classes.^16^ Participants were classified into two groups: physically inactive (engaged in no PA or between 1 and 299 minutes per week), or physicallyactive (≥300 minutes per week).

Sedentary behavior was assessed by the question: “How many hours per day do you usually sit not only watching television, but doing other activities such as using a computer, playing video games, or doing other activities while sitting down? (disregarding Saturdays, Sundays, holidays, or time spent sitting at school)”.^16^ The variable was organized into “Less than 4 hours a day”, and “≥4 hours a day.”

Smoking history or present smoker was assessed by the question: “Do you smoke cigarettes?”, given the options “No,” “Yes, at least one cigarette last month,” “Former smoker,” and “Do not know or Do not want to respond.” This variable was dichotomized into No (No/Former smoker/Do not know or Do not want to respond) and Yes (At least one cigarette last month). History of alcohol consumption (called binge drinking) was defined as consuming ≥5 doses of alcohol for boys or ≥4 doses for girls at least one day over the past 30 days, being dichotomized into presence or absence of a history of binge drinking.^17^

Sleep quality was assessed by asking: “How do you consider the quality of your sleep at the moment?”, with five possible responses (very good, good, regular, poor, and very poor). For later analysis, the categories “good and very good” and “regular, poor, and very poor” were grouped and transformed into a dichotomous variable containing “Good sleep quality” vs. “Poor sleep quality,” respectively.

The data were described using absolute and relative frequencies for the study’s categorical variables and means and standard deviations (SD) for the continuous variables. Fisher’s exact test was used to test associations between the three mental health symptoms in students with or without PSU. A Poisson regression model with robust variance was used to identify the associations of PSU with each symptom of mental health (depression, anxiety, and stress). This analysis was adjusted for the following variables: sex, age, skin color, SES, physical inactivity, smoking habit, binge drinking, sedentary behavior, and sleep quality. Prevalence ratios (PR) and 95% confidence intervals(95%CI) were used, with p-values <0.05 considered statistically significant for two-tailed tests. The data were analyzed using the STATA software (version 18.0).

## RESULTS

Of the 480 eligible students, 434 comprised the final study sample, yielding a participation rate of 90.4%. Most students were boys. The mean age was 17.2±1.5 years, ranging from 15 to 22 years. Most were 18 years or older, and most reported white skin color. Proportions of behavioral characteristics, including physical activity, cigarette smoking, history of binge drinking, sedentary behavior, sleep quality, and PSU, are presented in Table 1.

**Table 1:** Sociodemographic and behavioral characteristics of the sample of high school students in southern Brazil (n=434).

Characteristics	n	%
Sex		
Boys	238	55.2
Girls	193	44.8
Age (years)		
15 to 17	201	47.5
≥18	222	52.5
Skin color		
White	329	76.2
Other	103	23.8
Socioeoconomic status (quartile)		
1st	109	25.1
2nd	108	24.9
3rd	109	25.1
4th	108	24.9
Physically inactive		
Yes	136	31.5
Smoking habit		
Yes	23	5.3
Binge drinking		
Yes	155	36.7
Sedentary behavior (hours/day)		
<4	229	52.9
≥4	204	47.1
Sleep quality		
Poor	291	67.5
Good	140	32.5
Problematic smartphone use		
Yes	154	35.5

Source: Created by the authors.

In Table 2, we show the prevalence of each mental health symptom and the proportion of students with and without PSU. The prevalence values of symptoms of depression, anxiety, and stress were 37.1%, 44.7%, and 45.7%, respectively. In addition, almost two-thirds of the students (62.7%) had at least one mental health symptom. The proportion of mental health symptoms among students with PSU was 44.8% for anxiety, 57.1% for depression, and 58.2% for stress, totaling 76.0% who presented at least one of the mental health symptoms.

**Table 2:** Prevalence of mental health outcomes and the proportion of highschool students in southern Brazil with and without problematic smartphone use, 2022 (n=434).

Variables	Total n (%)	No PSU %	PSU %	p-value*
Symptoms of anxiety				
No	271 (63.9)	67.0	55.2	0.017
Yes	160 (37.1)	33.0	44.8	
Symptoms of depression				
No	240 (55.3)	62.0	42.9	<0.001
Yes	194 (44.7)	38.0	57.1	
Symptoms of stress				
No	235 (54.3)	60.9	41.8	<0.001
Yes	198 (45.7)	39.1	58.2	
Symptoms of at least one disorder				
No	162 (37.3)	44.4	24.0	<0.001
Yes	272 (62.7)	55.6	76.0	

PSU: Problematic Smartphone Use.

*Fisher’s Exact Test.

Source: Created by the authors.

In the crude analysis, all three symptoms and the presence of at least one of them were associated with PSU; however, in the adjusted analysis, only symptoms of depression and stress, in addition to the presence of at least one of the symptoms, had a 26%, 34%, and 24% higher prevalence, respectively, in the group of students with PSU, compared to those without PSU (Table 3).

**Table 3:** Associations of mental health outcomes with problematic smartphone use among high school students in southern Brazil, 2022 (n=434).

Variables	PSU					
	Crude analysis		Adjusted analysis*	
	PR	95%CI	PR	95%CI
Symptoms of anxiety				
No	1.00		1.00	
Yes	1.36	1.07–1.73	1.09	0.85–1.40
Symptoms of depression				
No	1.00		1.00	
Yes	1.50	1.23–1.84	1.26	1.02–1.56
Symptoms of stress				
No	1.00		1.00	
Yes	1.49	1.22–1.82	1.34	1.09–1.64
Symptoms of at least one disorder				
No	1.00		1.00	
Yes	1.37	1.19–1.56	1.24	1.08–1.43

PSU: Problematic Smartphone Use; PR: Prevalence ratio; 95%CI: 95% confidence interval.

*Adjusted by: sex, age, skin color, socioeconomic status, physical inactivity, smoking habit, binge drinking, sedentary behavior, and sleep quality.

Source: Created by the authors.

## DISCUSSION

In this study, we analyzed the association between PSU and symptoms of anxiety, depression, and stress among high school students in the extreme south of Brazil. According to the results, about one-third of them were classified as having PSU. More than half presented symptoms of anxiety, depression, or stress. PSU was associated with a higher prevalence of mental health symptoms.

In addition, it is worth mentioning that, globally, individuals aged 10–24 years account for 25% of all mental disorders across all age groups.^18^ Furthermore, the rates of disability-adjusted life years (DALYs) among youth have recently increased,^18^ with anxiety and depressive disorders showing the highest growth in DALYs between 2010 and 2023 among all causes.^19^

The results of this study are consistent with evidence from systematic reviews and meta-analyses involving populations from other countries, which also observed significant associations between maladaptive use of smartphones and mental health problems in young people.^9,12^ It is worth noting, however, that although different instruments were used to assess PSU, the prevalence of this behavior identified in the present study was lower than that observed in Taiwan (55%) and Turkey (50%),^20,21^ considered developing countries, and higher than the prevalence found in Korea (25%) and Switzerland (16%), considered developed countries.^22,23^ Moreover, with different ways to assess maladaptive use of smartphones, in Brazil, there is evidence that the prevalence may range from 53.3 to 70.3% among young people of the Southeast, Midwest, and Northeast regions of the country,^12,15,24^ underscoring the importance of conducting an initial screening to identify its presence across distinct sociocultural contexts.

In the present study, we observed that young people with PSU were more likely to have depressive symptoms, in line with findings from previous studies.^3^ Depression in young people is often characterized by depressed mood most of the day, loss of interest or pleasure in previously rewarding activities, sleep and appetite disturbances, fatigue, feelings of worthlessness or guilt, impaired concentration, psychomotor retardation, and recurrent suicidal ideation.^25^ In the context of mobile device use, excessive use often reduces the time devoted to face-to-face interactions or outdoor activities, practices that are positively associated with better mental health outcomes.^26^ This replacement of face-to-face activities with the digital environment tends to intensify feelings of loneliness, isolation, and negative affect.^3,7,9^Finally, PSU can disrupt sleep patterns, a factor that has a robust and well-documented association with depression.

Stress among young people is a natural response of the body to physical, emotional, or mental pressures.^27^ In the present study, 45% of young people with PSU also reported stress symptoms, an association similarly corroborated by Xu et al.^28^ This phenomenon can be explained by the search for homeostatic balance, considering that the use of the device is associated with pleasure and has addictive potential.^28^ Furthermore, the continuous flow of notifications from smartphones, coupled with social pressure for aesthetic and behavioral standards imposed by the Internet, creates an environment conducive to stress. In addition, the preference for using the device over productive activities can lead to procrastination, which raises stress levels.^9,12^

Conversely, anxiety can be understood as a “state of alertness,” characterized by a set of physiological and behavioral reactions associated with situations of fear or anticipation of danger.^29^ Although unpleasant, the manifestation of anxious symptoms is considered an expected response of the individual in the face of adversity. Unlike previous investigations, in which anxiety was identified as a mediating variable between stress and depressive symptoms,^3,28^ in the present study, we found no statistically significant association between anxiety and PSU in the adjusted analysis. Notably, this association was significant in the crude analysis but lost significance after adjustment, suggesting that confounding factors, particularly sleep quality, which affected 67.5% of the sample, may have accounted for the observed crude association. This is consistent with evidence showing that sleep mediates the relationship between screen time and mental health outcomes in adolescents.^7^ Furthermore, authors of meta-ana-lytic reviews have reported small effect sizes and substantial heterogeneity for the PSU–anxiety association, with low-quality evidence (GRADE) supporting this link.^9^ One additional hypothesis lies in the highly demanding academic profile of federal institutes, where chronic stress is normalized and the high baseline prevalence of anxiety (44.7%), the highest among

the three symptoms, may reflect shared environmental stressors that affect students regardless of PSU status. In this context, smartphones may serve as an avoidant or compensatory coping strategy rather than a direct cause of anxiety.^30^

This study had some limitations. Firstly, it is not possible to establish causality in the associations found due to the study’s design, as it is not possible to know whether the participants already had mental health problems before PSU. Secondly, the study has limited external validity, as it is not a representative sample of high school students from the entire municipality. However, it should be noted that research conducted in schools provides important insights for managers to develop projects and public policies to reduce risk factors to students’ health and integrity. Among the strengths, we highlight the assessment of main variables by two validated instruments for use with schoolchildren (Smartphone Addiction Scale – Short Version and the DASS-21), and the fact that the study presents data related to PSU and mental health symptoms in a population of young people in Brazil, as there are few published studies on the subject.

In conclusion, PSU was associated with a higher prevalence of symptoms of mental health in high school students in the south of Brazil, especially depression and stress. These results contribute to the growing literature investigating the association between PSU and mental health, providing evidence that can guide clinical practices and public health policies aimed at promoting mental health in an increasingly connected world.

## Data Availability

The database that originated the article is available with the corresponding author.

## References

[1] Nawaz S (2024). Distinguishing between effectual, ineffectual, and problematic smartphone use: a comprehensive review and conceptual pathways model for future research. Comput Hum Behav Rep.

[2] Nawaz S, Bhowmik J, Linden T, Mitchell M (2025). Exploring the impact of smartphone dependency on real-life recreational activities: a theory of planned behaviour study. Entertain Comput.

[3] Elhai JD, Yang H, Montag C (2021). Fear of missing out (FOMO): overview, theoretical underpinnings, and literature review on relations with severity of negativeaffectivity and problematic technology use. Braz J Psychiatry.

[4] Turner A [homepage on the Internet] How many people have smartphones worldwide.

[5] Nery C (2024). Internet foi acessada em 72, 5 milh”es de domicàlios do paàs em 2023.

[6] Panova T, Carbonell X (2018). Is smartphone addiction really an addiction?. J Behav Addict.

[7] Santos RM, Mendes CG, Sen Bressani GY, Ventura SA, Nogueira YJ, Miranda DM (2023). The associations between screen time and mental health in adolescents: a systematic review. BMC Psychol.

[8] Neves AB, Rocha AG, Neves IC, Melo AM (2020). Associa?¡o entre uso nocivo das redes sociais e a depress¡o em adolescentes: uma revis¡o sistemøtica. Rev Psicol.

[9] Sohn SY, Rees P, Wildridge B, Kalk NJ, Carter B (2019). Prevalence of problematic smartphone usage and associated mental health outcomes amongst children and young people: a systematic review, metaanalysis and GRADE of the evidence. BMC Psychiatry.

[10] Liu W, Zhang Y, Chen J, Li X, Huang Y, Zhao F (2025). Global burden and trends of major mental disorders in individuals under 24 years of age from 1990 to 2021, with projections to 2050: insights from the Global Burden of Disease Study 2021. Front Public Health.

[11] World Health Organization [homepage on the Internet] Addressing the digital determinants of youth mental health and well-being: policy brief.

[12] Nunes PP, Abdon AP, Brito CB, Silva FV, Santos IC, Martins DD (2021). Fatores relacionados æ depend»ncia do smartphone em adolescentes de uma regi¡o do Nordeste brasileiro. Ci»nc Saÿde Colet.

[13] Patias ND, Machado WL, Bandeira DR, Dell’Aglio DD (2016). Depression Anxiety and Stress Scale (DASS-21) – Short Form: adaptaç¡o e validaç¡o para adolescentes brasileiros. Psico-USF.

[14] Martins BG, Silva WR, Maroco J, Campos JA (2019). Escala de Depress¡o, Ansiedade e Estresse: propriedades psicom«tricas e preval»ncia das afetividades. J Bras Psiquiatr.

[15] Andrade AL, Scatena A, Martins GG, Pinheiro BO, Silva AB, Enes CC (2020). Validation of smartphone addiction scale – Short version (SAS-SV) in Brazilian adolescents. Addict Behav.

[16] Instituto Brasileiro de Geografia e Estatàstica (2021). Pesquisa nacional de saÿde do escolar: 2019.

[17] Siqueira L, Smith VC (2015). Committee on Substance Abuse. Binge drinking. Pediatrics.

[18] Wang Z, Dou Y, Yang X, Guo X, Ma X, Zhou B (2025). Global, regional, and national burden of mental disorders among adolescents and young adults, 1990–2021: a systematic analysis for the Global Burden of Disease Study 2021. Transl Psychiatry.

[19] GBD 2023 Disease and Injury and Risk Factor Collaborators (2025). Burden of 375 diseases and injuries, risk-attributable burden of 88 risk factors, and healthy life expectancy in 204 countries and territories, including 660 subnational locations, 1990–2023: a systematic analysis for the Global Burden of Disease Study 2023. Lancet.

[20] Liu CH, Lin SH, Pan YC, Lin YH (2016). Smartphone gaming and frequent use pattern associated with smartphone addiction. Medicine (Baltimore).

[21] F-rat S, Gül H, Sertçelik M, Gül A, Gürel Y, K-l-ç BG (2018). The relationship between problematic smartphone use and psychiatric symptoms among adolescents who applied to psychiatry clinics. Psychiatry Res.

[22] Jeong A, Ryu S, Kim S, Park HK, Hwang HS, Park KY (2023). Association between problematic smartphone use and physical activity among adolescents: a path analysis based on the 2020 korea youth risk behavior web-based survey. Korean J Fam Med.

[23] Haug S, Castro RP, Kwon M, Filler A, Kowatsch T, Schaub MP (2015). Smartphone use and smartphone addiction among young people in Switzerland. J Behav Addict.

[24] Freitas BH, Gaàva MA, Diogo PM, Bortolini J (2022). Relationship between Lifestyle and Self-Reported Smartphone Addiction in adolescents in the COVID-19 pandemic: a mixed-methods study. J Pediatr Nurs.

[25] American Psychiatric Association (2013). Diagnostic and statistical manual of mental disorders: DSM-5^TM^.

[26] Kushlev K, Dwyer R, Dunn EW (2019). The social price of constant connectivity: smartphones impose subtle costs on well-being. Curr Dir Psychol Sci.

[27] Gee DG, Sisk LM, Cohodes EM, Bryce NV (2022). Leveraging the science of stress to promote resilience and optimize mental health interventions during adolescence. Nat Commun.

[28] Xu TT, Wang HZ, Fonseca W, Zimmerman MA, Rost DH, Gaskin J (2019). The relationship between academic stress and adolescents’ problematic smartphone usage. Addict Res Theory.

[29] Barlow DH, Durand VM (2015). Psicopatologia: uma abordagem integrada.

[30] Kardefelt-Winther D (2014). Comput Human Behav.

